# Envelope: interactive software for modeling and fitting complex isotope distributions

**DOI:** 10.1186/1471-2105-9-446

**Published:** 2008-10-20

**Authors:** Michael T Sykes, James R Williamson

**Affiliations:** 1 Department of Molecular Biology, The Scripps Research Institute, La Jolla, California 92037, USA; 2 Department of Chemistry, The Scripps Research Institute, La Jolla, California 92037, USA; 3 Skaggs Institute for Chemical Biology, The Scripps Research Institute, La Jolla, CA 92037, USA

## Abstract

**Background:**

An important aspect of proteomic mass spectrometry involves quantifying and interpreting the isotope distributions arising from mixtures of macromolecules with different isotope labeling patterns. These patterns can be quite complex, in particular with *in vivo *metabolic labeling experiments producing fractional atomic labeling or fractional residue labeling of peptides or other macromolecules. In general, it can be difficult to distinguish the contributions of species with different labeling patterns to an experimental spectrum and difficult to calculate a theoretical isotope distribution to fit such data. There is a need for interactive and user-friendly software that can calculate and fit the entire isotope distribution of a complex mixture while comparing these calculations with experimental data and extracting the contributions from the differently labeled species.

**Results:**

Envelope has been developed to be user-friendly while still being as flexible and powerful as possible. Envelope can simultaneously calculate the isotope distributions for any number of different labeling patterns for a given peptide or oligonucleotide, while automatically summing these into a single overall isotope distribution. Envelope can handle fractional or complete atom or residue-based labeling, and the contribution from each different user-defined labeling pattern is clearly illustrated in the interactive display and is individually adjustable. At present, Envelope supports labeling with ^2^H, ^13^C, and ^15^N, and supports adjustments for baseline correction, an instrument accuracy offset in the m/z domain, and peak width. Furthermore, Envelope can display experimental data superimposed on calculated isotope distributions, and calculate a least-squares goodness of fit between the two. All of this information is displayed on the screen in a single graphical user interface. Envelope supports high-quality output of experimental and calculated distributions in PNG or PDF format. Beyond simply comparing calculated distributions to experimental data, Envelope is useful for planning or designing metabolic labeling experiments, by visualizing hypothetical isotope distributions in order to evaluate the feasibility of a labeling strategy. Envelope is also useful as a teaching tool, with its real-time display capabilities providing a straightforward way to illustrate the key variable factors that contribute to an observed isotope distribution.

**Conclusion:**

Envelope is a powerful tool for the interactive calculation and visualization of complex isotope distributions for comparison to experimental data. It is available under the GNU General Public License from .

## Background

**Figure 1:**
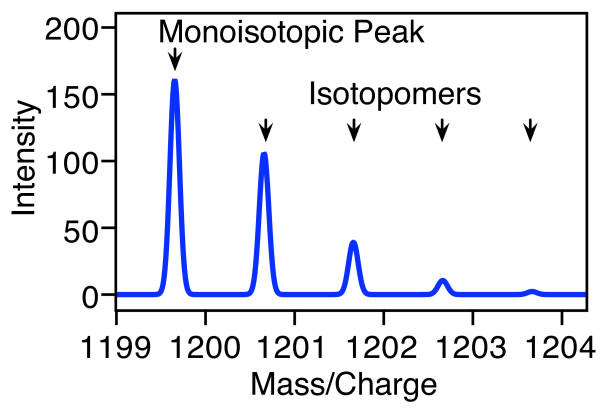
**A calculated isotope distribution**. The mass spectrum of a peptide or oligonucleotide consists of a statistical distribution of peaks that arises from the natural abundance of heavy isotopes. The calculated spectrum of the unlabeled peptide NVLPQRSTVW shown here consists of a monoisotopic peak at the lowest mass/charge value, and several additional peaks at higher mass/charge values. These additional peaks are heavy isotopomers that result from the natural abundance isotopes.

Mass spectrometry (MS) is an increasingly important technique in proteomic research, providing insights into protein expression, turnover and metabolism [1]. When an organism growing in unlabeled medium is supplied with a pulse of isotopically labeled nutrients such as ammonium ions, glucose or amino acids, metabolic labeling of the proteome occurs, resulting in a mixture of both unlabeled and labeled cellular components. Alternatively, two or more samples may be independently labeled and then mixed together for analysis, yielding mass spectra with a combination of unlabeled and labeled species [2]. Even the mass spectrum of a single unlabeled peptide can be quite complex, due to the natural abundance of heavy isotopes. The small fraction of naturally labeled atoms creates a statistical distribution of peaks rather than just a single peak, as illustrated in Figure 1. Several other factors can increase the complexity of the observed spectrum, such as the presence of a number of species with different labeling patterns, each with their own distribution of peaks, or the extent of labeling that is achieved. Partial labeling compounds the complexity of the mass spectrum by broadening the statistical distribution of peaks for a species. This is especially relevant to the study of higher organisms, where it can be difficult to achieve high extents of labeling [3-5].

One of the challenges of quantitative mass spectrometry is dealing with this rich and complex distribution of peaks. Several software packages exist for the analysis of MS data, including OpenMS [6], MapQuant [7], MASPECTRAS [8], msInspect [9], MzMine [10], SpecArray [11], TPP [12], Viper [13], Superhirn [14], XCMS [15], mMass [16] and isodist [17]. Each of these programs focuses on the high-throughput processing of large datasets, which is crucial to analyzing proteomic MS data. There is a more limited set of tools aimed at calculating and visualizing individual isotope distributions. These include iMass [18], Isotopica [19], MS-Isotope [20] and Isotopident [21]. However none of these tools are interactive, offer fitting of the entire isotope distribution, and a flexible definition of the labeling pattern.

Here, the program Envelope is described, offering a more sophisticated approach to the visualization of calculated isotope distributions than any existing software package. Any number of different labeling patterns and labeled species can be accommodated for both peptides and oligonucleotides. In addition, Envelope offers the ability to interact with data by directly comparing calculated distributions with experimental data. Envelope features a user-friendly interface, and the displayed isotope distributions change in near real-time in response to user-controlled changes in the labeling parameters using continuously variable slider controls or text input boxes. Envelope is a flexible tool designed to help researchers interpret the complex spectra they may see in their experiments, and to help plan new experiments by illustrating which combinations of labels will yield interpretable spectra. Envelope will also be useful in a classroom environment to help explain the principles of MS and isotope distributions since no scripting or command line knowledge is necessary. Envelope is a powerful MS analysis package that is well suited for a variety of research and educational purposes.

## Implementation

### Source Code and Algorithms

Envelope is an open source desktop application. The user interface and visualization components are written in Objective-C, taking advantage of the Mac OS X; Cocoa framework, while the isotope distribution calculation core is written in C. Isotope distributions are calculated using the Fourier Transform convolution (FTC) method previously described by Rockwood *et al *[22,23] and subsequently extended by Sperling *et al *[17]. Briefly, MS datasets have the mass/charge (m/z) ratio as the independent variable (*m*-domain), and intensity as the dependent variable. A spectrum of *N *real points in the *m*-domain *S*(*m*) has a conjugate Fourier representation as a frequency in the *μ*-domain *s*(*μ*), where the *μ*-domain representation is complex. The two representations can be interconverted by forward Fourier Transforms (FT) and inverse Fourier Transforms (IFT). The isotope distribution for a species with a particular labeling pattern is first calculated in the *μ*-domain, followed by IFT to obtain the *m*-domain spectrum. FTC was chosen over the polynomial method [24] as it is an exact method, and lends itself well to the calculation of both atom-based and residue-based labeling patterns [17]. Fourier Transforms were implemented using the FFTW library [25]. The Envelope executable (Additional file 1) and source code (Additional file 2) are both available for download as additional files or from the Envelope website.

### Features

Envelope is capable of simultaneously calculating the isotope distributions for any number of different species for a single peptide or oligonucleotide, and experimental labeling patterns of virtually any level of complexity can be handled. Calculated spectra for each of the different labeling patterns specified can be visualized individually or all at the same time, and a spectrum containing the sum of all distributions (*S*_*Tot*_(*m*)) is automatically calculated.

$$S_{Tot}(m)=\sum_{i=1}^{n}A_{i}S{\left(m\right)}_{i}$$

The isotope distribution for a species with a particular labeling pattern has a unit spectrum *S*(*m*)_*i *_as well as an amplitude *A*_*i *_by which the unit spectrum is multiplied. The labeling patterns are defined in terms of fractional ^2^H, ^13^C, and ^15^N content, and can be further defined in terms of all atoms of a particular type for the entire molecule (atom-based labeling) or only atoms belonging to specific residue types (residue-based labeling). In order to account for hydrogen exchange, two categories of hydrogen labeling can be defined for subsets of a residue's hydrogen atoms, each with an independent fraction of ^2^H. Hydrogen atoms beyond the sum of these two categories are assumed to have natural abundance isotope content.

Envelope can concurrently load multiple experimental data files that contain pairs of m/z and intensity values. These data are displayed with the calculated spectra in the main window, and the goodness of fit of each data set to the sum of all the calculated spectra is automatically calculated using the reduced chi-squared (χ^2^) formula.

$$\chi^{2}=\frac{1}{n}\sum_{i=1}^{n}\frac{{\left(I_{i,data}-I_{i,calc}\right)}^{2}}{\sigma_{i}^{2}}$$

The average of the squares of the residuals between the experimental intensity *I*_*data *_and the calculated intensity *I*_*calc *_is summed over *n *experimental data points, while the error estimate σ is arbitrarily set to one for convenience. Chi-squared is best used as a relative quantity to compare multiple fits to a single experimental spectrum. It is difficult to directly compare fits to different experimental spectra using chi-squared as it depends on several factors including overlapping peaks and the intensity of the acquired spectra. To obtain a better fit to the experimental data, adjustments can be made to the baseline, peak width and mass offset of a calculated spectrum. The mass offset is a uniform offset in the m/z domain that is applied as a small mass accuracy correction. The Gaussian peak width γ is defined in the μ domain by the following function where smaller values of γ lead to broader peaks.

$$g(\mu )=\frac{\exp\left(-\mu^{2}/2\gamma^{2}\right)}{\sqrt{2}\pi \gamma }$$

**Figure 2:**
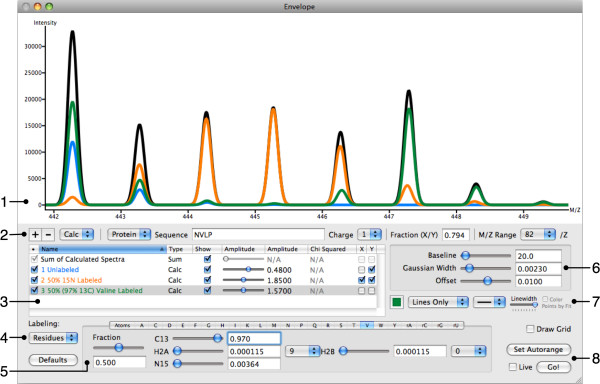
**The Envelope user interface**. (1) The main display for isotope distributions. Users can zoom (click and drag), move the display (control-clicking and drag), or reset the zoom level (double-click). (2) Add or remove spectra with the "+" and "-" buttons. A drop down menu selects for protein or RNA, and controls define both sequence and charge. "Fraction" is a user-defined quantity determined by the amplitudes of selected calculated spectra. The M/Z Range menu allows the user to specify a greater mass range for large peptides. (3) A list of all spectra, each with controls to hide or display its trace and adjust its amplitude. By deselecting the checkbox in the leftmost column (•), calculated spectra are disabled. The fraction from (2) is defined here via checkboxes X and Y, and chi-squared is displayed for experimental data. (4) Choose between unlabeled, atom-based labeling, or residue-based labeling for the species currently selected in (3). (5) Define the labeling pattern for the species currently selected in (3). Displayed is the definition for valine (residue-based labeling), which is 50% labeled with 97% ^13^C. For individual residues the amount of ^13^C or ^15^N can be specified, as well as the fraction of the residue that is labeled. Two different ^2^H values can be defined and applied to a subset of the residue's hydrogen atoms to account for hydrogen exchange. Hydrogens beyond those two groups are assumed to have natural abundance. (6) Global adjustments for baseline, Gaussian width and offset. These apply to all calculated spectra and can be adjusted to fit experimental data. (7) Change the appearance of traces. (8) Initiate calculation of spectra or auto-zoom. If the "Live" checkbox is selected, spectra will automatically update in response to user-initiated changes. Displayed are species for the peptide NVLP; unlabeled (blue), 50% ^15^N labeled (orange) and containing 50% labeled valine, itself 97% ^13^C labeled. The sum of these species is displayed in black.

Both calculated and experimental spectra can be displayed as a combination of lines and/or points with user-definable colors and styles. The range of data displayed is automatically optimized, although the user can manually zoom in to examine specific areas. Envelope is capable of high-quality PDF and PNG output, allowing for the easy generation of publication-quality images. The user interface shown in Figure 2 is dominated by the graphical display, and a second region contains the bulk of the controls.

It would be useful for Envelope to be able to interact with the wide variety of existing MS analysis programs. To this end a subset of Envelope's functionality can be manipulated by script, allowing other programs to use Envelope as a frontend for the display of calculated and experimental isotope distributions.

### Usage

Upon opening, Envelope presents the user with the default peptide NVLP, and two entries in the list of spectra, one entry for a calculated spectrum with natural abundance isotope levels, and one entry for the sum of all calculated spectra, which cannot be deleted. Pressing the Go button at this point will display the natural abundance isotope distribution for the peptide NVLP, z = +1. The Go button may be activated at any point to visualize changes that have occurred, or the Live checkbox may be selected and spectra will automatically be recalculated in response to user-initiated changes. The user may add additional calculated spectra using the "+" button, and the labeling pattern may be defined at any time. Only one calculated spectrum is needed to begin visualizing isotope distributions, though a distinct calculated spectrum is required for each species generated in an experiment. In quantitative proteomics experiments, there are typically one unlabeled and at least one labeled species. Once the necessary calculated spectra have been added, experimental data can be loaded by the "+" button, or via the File menu. The sequence, molecule type and charge will each need to be adjusted to match the data. The user may then adjust the labeling patterns for the calculated species, their amplitudes, and the baseline, Gaussian width and offset in order to fit the sum of the calculated spectra to the data by monitoring the decrease in χ^2^. Envelope will display any desired ratio of amplitudes of calculated spectra defined by the user in order to quantitatively compare the amounts of different species. This value dynamically updates during the fitting process, always reflecting the current amplitudes.

## Results

### ^15^N Pulse Labeling

**Figure 3:**
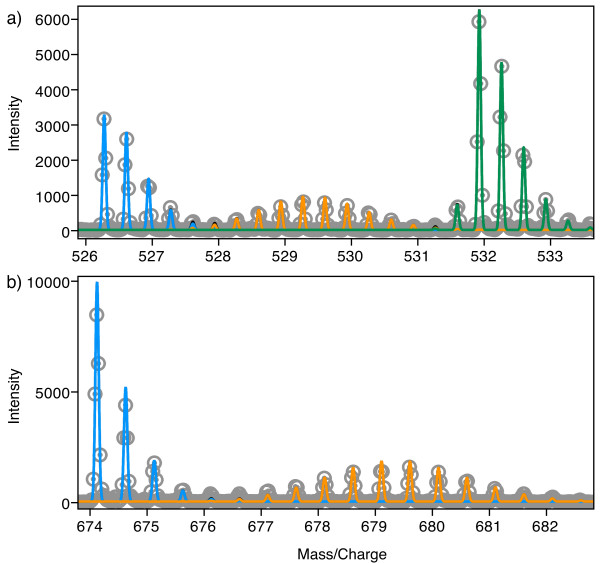
**Atom-based labeling with ^15^N**. a) The mass spectrum for ribosomal protein S2, residues 45–58 (TVPMFNEALAELNK, z = +3). Experimental data points are shown as grey circles with centered dots. The calculated unlabeled isotope distribution is shown in blue, the partially labeled distribution (50% ^15^N) shown in orange, and the fully labeled distribution (99.3% ^15^N) shown in green. b) The isotope distribution for the 16S RNA, nucleotides 766–769 (AAAG, z = -2). Experimental data points are shown as grey circles with centered dots. The calculated unlabeled isotope distribution is shown in blue and the partially labeled (50% ^15^N) distribution is shown in orange. In both cases, the different labeled species were fit by hand to match the experimental data using Envelope, and the figures were directly exported from Envelope. The data used is available for download (Additional file 3).

To illustrate the features of Envelope, a complex isotope distribution was generated using peptide samples with a combination of partial metabolic ^15^N labeling and uniform ^15^N labeling. A rapidly growing culture of *E. coli *in medium with (^14^NH_4_)_2_SO_4 _as the sole nitrogen source was pulsed with (^15^NH_4_)_2_SO_4_, to a final enrichment of ~50% ^15^N. After approximately 20 minutes of continued growth, ribosomes were harvested from the cells, producing a mixture of unlabeled and 50% ^15^N labeled ribosomes. Fully ^15^N labeled ribosomes (99.3% ^15^N) were then added to the mixture, resulting in three species for each protein: unlabeled protein (synthesized before the pulse), partially ^15^N labeled protein (synthesized after the pulse) and fully ^15^N labeled protein (added externally). The ribosomal proteins were purified, and the mixture was digested with trypsin and analyzed by liquid chromatography coupled mass spectrometry (LC/MS) using an ESI-TOF instrument (Agilent). The peaks resulting from a single peptide (protein S2, residues 45–58, TVPMFNEALAELNK, z = +3) are shown in Figure 3a. The three distinct species with different isotope labeling patterns are clearly observable in the experimental data, and using Envelope three calculated distributions can be interactively fit by hand to the experimental data. Each of these three peptide species is defined by the user, in this case using atom-based labeling to specify the ^15^N content. The amplitudes of the unlabeled, partially labeled and fully labeled species (0.260, 0.175 and 0.480 respectively) give information about the relative quantities of the three different species present in the mixture for the protein S2. The fraction labeled (*f*) is defined as the amplitude of the partially metabolically labeled species (*A*_*L*_) divided by the sum of the amplitudes of the unlabeled (*A*_*U*_) and metabolically labeled species.

$$f=\frac{A_{L}}{A_{U}+A_{L}}$$

For this peptide *f *= 0.402, indicating that approximately 40% of the S2 protein in the sample was labeled with 50% ^15^N.

In a similar metabolic labeling experiment the ribosomal RNA was purified, digested with RNAse T1 and submitted for LC/MS analysis in negative ion mode, without the addition of a fully ^15^N labeled species. The result is a mixture of just two species, one unlabeled and the other partially labeled (50% ^15^N). The experimental spectrum resulting from these two species, as well as the calculated isotope distribution (16S RNA, residues 766–769, AAAG, z = -2) are shown in Figure 3b. The amplitudes of the unlabeled and partially labeled species are 0.550 and 0.350 respectively, and *f *= 0.389. The data used is available for download (Additional File 3).

### Pulse Labeling with Amino Acids

**Figure 4:**
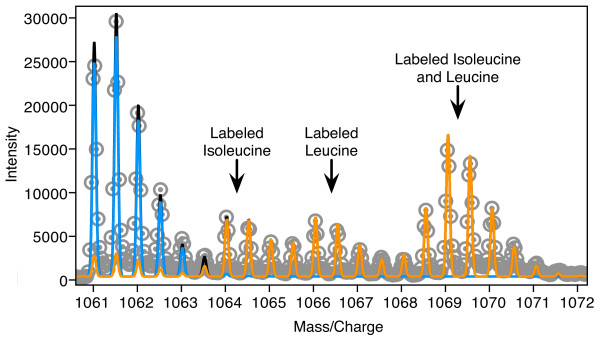
**Residue-based labeling with ^2^H-Leu and ^13^C-Ile**. The mass spectrum for ribosomal protein S4, residues 204–224 (GEILGGMAAVEQPEKPAAQPK, z = +2). Shown in grey are experimental data points. In blue is the calculated distribution for the unlabeled species, in orange the calculated distribution for the labeled species, and in black the sum of the two. The labeled species is composed of 72% isoleucine labeled with 97% ^13^C, and 72% leucine labeled with 97% ^2^H_9 _and 85% ^2^H_1 _(due to hydrogen exchange). These enrichment levels were determined empirically using Envelope. In the case of the extent of amino acid enrichment, values dictated by the experimental protocol were used as a starting point. Four distinct clusters of peaks are observed in the spectrum resulting from peptides which have no labeled amino acids, a single labeled isoleucine, a single labeled leucine, or both a labeled isoleucine and a labeled leucine. Note that there is a small but significant contribution to the unlabeled peak from the fractionally labeled species (orange). This contribution is due to partial labeling with both leucine and isoleucine (72%), resulting in a statistical subset of labeled peptides that do not contain either a labeled leucine or a labeled isoleucine. The amplitude of the unlabeled species (blue) must be adjusted so that the sum of the two contributions (black) matches the experimental spectrum. The data used is available for download (Additional File 3).

In addition to calculating isotope distributions for species with atom-based labeling, Envelope is also capable of calculating distributions for species that are labeled with amino acids or nucleotides. Rapidly growing *E. coli *in minimal medium supplemented with each of the 20 amino acids was simultaneously pulsed with both ^13^C labeled isoleucine and ^2^H labeled leucine, to a final ratio of approximately 3:1 labeled:unlabeled amino acids. Ribosomes were harvested and ribosomal proteins purified after an additional 25 minutes of growth. The result was a mixture with two species, one unlabeled species and one species fractionally labeled with both ^2^H leucine and ^13^C isoleucine. The experimental spectrum for one peptide (protein S3, residues 204–224, GEILGGMAAVEQPEKPAAQPK, z = +2) along with the calculated isotope distributions for the two species is shown in Figure 4. Since this peptide has just one leucine residue and one isoleucine residue, there are four identifiable groups of peaks, resulting from all four possible combinations of labeled residues: no labeled amino acids, labeled isoleucine only, labeled leucine only, and both labeled leucine and isoleucine. The relative intensities of these four groups of peaks are determined by the fraction of labeled leucine and isoleucine residues in the labeled species, which was observed to be ~72% for both amino acids. This value was determined empirically using Envelope to fit the experimental spectrum, starting from enrichment levels expected based on the experimental protocol. The amplitudes of the unlabeled and labeled species are 2.60 and 3.20 respectively, and *f =*0.552. The data used is available for download (Additional File 3).

## Discussion

While Envelope is not intended to replace high-throughput batch analysis programs for the large-scale fitting of data in a research environment, it has its own place alongside them. Envelope is useful in planning experiments by visualizing the isotope distributions produced by potential labeling patterns. By exploring the predicted isotope distributions before performing any actual experiments, optimal labeling patterns can be determined which help increase the quality of the final data. Envelope also allows the user to fit calculated distributions to experimental spectra that may be poorly fit by automatic fitting routines. This is especially true in the case of spectral overlap, where to the human eye it may be clear that adjacent peaks are overlapping the signal from the peptide of interest, but a computer algorithm cannot resolve this situation. A least-squares fit for example will increase the baseline of the calculated spectrum or amplitude of a species in an attempt to fit overlapping experimental peaks where no calculated distribution exists, skewing the final result. In a case where there is an unknown extent of isotope enrichment, Envelope can be used to fit data for a few peaks by hand, determining the actual enrichment to be used as a starting value for a batch fit using another program.

More than just a research tool, Envelope is useful as a learning aid, both for individual researchers and in the classroom environment. By defining a labeling pattern and immediately seeing the calculated distribution onscreen, then watching the distribution change in response to adjustments to the labeling pattern, one can easily understand the different factors that contribute to a given isotope distribution. There are two important concepts that must be distinguished in metabolic labeling experiments, which are the concepts of fraction labeled (*f*), defined here as the relative amount of the labeled species compared to the unlabeled species, and the isotope abundance which is the fractional isotope content of a particular labeled species. For students being introduced to the subject for the first time, these two distinct quantities can be confusing as they both deal with a different kind of extent of labeling. Using Envelope, these two concepts can be directly illustrated and explored as a didactic exercise. Envelope features slider bars to manipulate the isotope enrichment for a species while observing the effect on the spectrum in the interactive display, and checkboxes to define the fraction labeled in terms of the amplitudes of different species. These changes are displayed in near real-time, dependent on processor speed and complexity of the system. This immediate feedback is very useful to illustrate how the parameters affect the isotope distribution, and has been very effective as a live tool in seminars describing the analysis of LC/MS data from metabolic labeling experiments.

## Conclusion

Envelope is a powerful tool for the interactive calculation and visualization of isotope distributions that is capable of simultaneously calculating distributions for an arbitrary number of species of a single peptide or oligonucleotide, each with a different labeling pattern. Envelope can visualize experimental mass spectra, allowing the user to perform manual least-squares fits of calculated distributions to real experimental data. Envelope is useful for small-scale data analysis and planning experiments. Moreover it can be used as a teaching tool, and its user-friendly and interactive qualities make it well suited for use by research groups, in seminars, or in the classroom.

## Availability and requirements

Project name: Envelope

Project home page: 

Operating system(s): Mac OS X; 10.4 or 10.5, Intel and Power PC

Programming language: C and Objective-C

Other requirements: Executable: None. Source: Compilation of the Envelope source requires the Apple Developer Tools, freely available from . Envelope makes use of the FFTW library, freely available under the terms of the GNU General Public License from .

License: GNU General Public License (GPL)

Any restrictions to use by non-academics: See GPL license for details

## Abbreviations

MS: Mass spectrometry; FTC: Fourier Transform convolution; FT: Fourier Transform; IFT: Inverse Fourier Transform; LC/MS: Liquid chromatography coupled mass spectrometry; ESI-TOF: Electrospray ionization time-of-flight.

## Authors' contributions

MTS designed and wrote the software and built the project home page. MTS and JRW both tested the software and contributed to the manuscript.

## Supplementary Material

Additional file 1 **zip archive of the Envelope application.** Click here for file

Additional file 2 **zip archive of the Envelope source code.** Click here for file

Additional file 3 **zip archive of sample data used in Figures **3** and **4. Click here for file

## References

[B1] Smith JC, Lambert J-P, Elisma F, Figeys D (2007). Proteomics in 2005/2006: developments, applications and challenges. Anal Chem.

[B2] Oda Y, Huang K, Cross FR, Cowburn D, Chait BT (1999). Accurate quantitation of protein expression and site-specific phosphorylation. PNAS.

[B3] Doherty MK, McLean L, Beynon RJ (2007). Avian proteomics: advances, challenges and new technologies. Cytogenet Genome Res.

[B4] Doherty MK, Whitehead C, McCormack H, Gaskell SJ, Beynon RJ (2005). Proteome dynamics in complex organisms: using stable isotopes to monitor individual protein turnover rates. Proteomics.

[B5] Hayter JR, Doherty MK, Whitehead C, McCormack H, Gaskell SJ, Beynon RJ (2005). The subunit structure and dynamics of the 20S proteasome in chicken skeletal muscle. Mol Cell Proteomics.

[B6] Sturm M, Bertsch A, Gröpl C, Hildebrandt A, Hussong R, Lange E, Pfeifer N, Schulz-Trieglaff O, Zerck A, Reinert K, Kohlbacher O (2008). OpenMS – an open-source software framework for mass spectrometry. BMC Bioinformatics.

[B7] Leptos KC, Sarracino DA, Jaffe JD, Krastins B, Church GM (2006). MapQuant: open-source software for large-scale protein quantification. Proteomics.

[B8] Hartler J, Thallinger GG, Stocker G, Sturn A, Burkard TR, Körner E, Rader R, Schmidt A, Mechtler K, Trajanoski A (2007). MASPECTRAS: a platform for management and analysis of proteomics LC-MS/MS data. BMC Bioinformatics.

[B9] Bellew M, Coram M, Fitzgibbon M, Igra M, Randolph R, Wang P, May D, Eng J, Fang R, Lin C, Chen J, Goodlett D, Whiteaker J, Paulovich A, McIntosh M (2006). A suite of algorithms for the comprehensive analysis of complex protein mixtures using high-resolution LC-MS. Bioinformatics.

[B10] Katajamaa M, Miettinen J, Oresic M (2006). MZmine: toolbox for processing and visualization of mass spectrometry based molecular profile data. Bioinformatics.

[B11] Li X-J, Yi EC, Kemp CJ, Zhang H, Aebersold R (2005). A software suite for the generation and comparison of peptide arrays from sets of data collected by liquid chromatography-mass spectrometry. Mol Cell Proteomics.

[B12] Keller A, Eng J, Zhang N, Li X-J, Aebersold R (2005). A uniform proteomics MS/MS analysis platform utilizing open XML file formats. Mol Syst Biol.

[B13] Monroe ME, Toliæ N, Jaitly N, Shaw JL, Adkins JN, Smith RD (2007). VIPER: an advanced software package to support high-throughput LC-MS peptide identification. Bioinformatics.

[B14] Mueller LN, Rinner O, Schmidt A, Letarte S, Bodenmiller B, Brusniak M-Y, Vitek O, Aebersold R, Müller M (2007). SuperHirn – a novel tool for high resolution LC-MS-based peptide/protein profiling. Proteomics.

[B15] Smith CA, Want EJ, O'Maille G, Abagyan R, Siuzdak G (2006). XCMS: processing mass spectrometry data for metabolite profiling using nonlinear peak alignment, matching, and identification. Anal Chem.

[B16] Strohalm M, Hassman M, Kosata B, Kodícek M (2008). mMass data miner: an open source alternative for mass spectrometric data analysis. Rapid Commun Mass Spectrom.

[B17] Sperling E, Bunner AE, Sykes MT, Williamson JR (2008). Quantitative analysis of isotope distributions in proteomic mass spectrometry using least-squares Fourier transform convolution. Anal Chem.

[B18] iMass. http://home.datacomm.ch/marvin/iMass/.

[B19] Fernandez-de-Cossio J, Gonzalez LJ, Satomi Y, Betancourt L, Ramos Y, Huerta V, Amaro A, Besada V, Padron G, Minamino N, Takao T (2004). Isotopica: a tool for the calculation and viewing of complex isotopic envelopes. Nucleic Acids Res.

[B20] MS-Isotope. http://prospector.ucsf.edu/.

[B21] Isotopident. http://www.isotopident.tk/.

[B22] Rockwood AL, van Orden SL, Smith RD (1995). Rapid Calculation of Isotope Distributions. Anal Chem.

[B23] Rockwood AL (1995). Relationship of Fourier Transforms to Isotope Distribution Calculations. Rapid Commun Mass Sp.

[B24] Yergey J, Heller D, Hansen G, Cotter RJ, Fenselau C (1983). Isotopic Distributions in Mass Spectra of Large Molecules. Anal Chem.

[B25] Frigo M, Johnson SG (2005). The design and implementation of FFTW3. P IEEE.

