# Supplementary material for: Envelope: interactive software for modeling and fitting complex isotope distributions
Source: BMC Bioinformatics. 2008 Oct 20;9:446. doi: 10.1186/1471-2105-9-446 (PMC2605472; doi:10.1186/1471-2105-9-446)
Supplement: Additional file 1 — zip archive of the Envelope application. [file 1471-2105-9-446-S1.zip › Envelope.app/Contents/Resources/Credits.html]

Envelope Credits


**Copyright:**

- Copyright 2008 Michael T. Sykes and James R. Williamson

  

**License:**

- GNU GPL

  

**Background Theory:**

- Rockwood, Van Orden and Smith. Rapid Calculation of Isotope Distributions, *Anal. Chem.*, **67**, 2699-2704 (1995).- Edit Sperling, Anne E. Bunner, Michael T. Sykes and James R. Williamson. Quantitative Analysis of Isotope Distributions in Proteomic Mass Spectrometry Using Least-Squares Fourier Transform Convolution. *Anal. Chem.*, **80**, 4906-4917 (2008).- isodist: Batch analysis using LS-FTC

  

**FT Library:**

- FFTW
