# Supplementary figures and images for: Envelope: interactive software for modeling and fitting complex isotope distributions
Source: BMC Bioinformatics. 2008 Oct 20;9:446. doi: 10.1186/1471-2105-9-446 (PMC2605472; doi:10.1186/1471-2105-9-446)

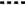

Supplement: Additional file 1 — zip archive of the Envelope application. [file 1471-2105-9-446-S1.zip › Envelope.app/Contents/Resources/dash1.png]

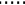

Supplement: Additional file 1 — zip archive of the Envelope application. [file 1471-2105-9-446-S1.zip › Envelope.app/Contents/Resources/dash2.png]

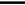

Supplement: Additional file 1 — zip archive of the Envelope application. [file 1471-2105-9-446-S1.zip › Envelope.app/Contents/Resources/line.png]

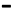

Supplement: Additional file 1 — zip archive of the Envelope application. [file 1471-2105-9-446-S1.zip › Envelope.app/Contents/Resources/minus.png]

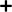

Supplement: Additional file 1 — zip archive of the Envelope application. [file 1471-2105-9-446-S1.zip › Envelope.app/Contents/Resources/plus.png]
