# Supplementary material for: Envelope: interactive software for modeling and fitting complex isotope distributions
Source: BMC Bioinformatics. 2008 Oct 20;9:446. doi: 10.1186/1471-2105-9-446 (PMC2605472; doi:10.1186/1471-2105-9-446)
Supplement: Additional file 2 — zip archive of the Envelope source code. [file 1471-2105-9-446-S2.zip › Envelope_src/CRC_2008_88th_Isotopes.pdf]

ATOMIC MASSES AND ABUNDANCES

This table lists the mass (in atomic mass units, symbol u) and the natural abundance (in percent) of the stable nuclides and a few important radioactive nuclides. A complete table of all nuclides may be found in Section 11 (“Table of the Isotopes”).

The atomic masses were taken from the 2003 evaluation of Audi, Wapstra, and Thibault (References 2, 3). The number in parentheses following the mass value is the uncertainty in the last digit(s) given. An asterisk * after an entry indicates the mass value was derived not purely from experimental data, but at least partly from systematic trends.

Natural abundance values were taken from the IUPAC Technical Report “Atomic Weight of the Elements: Review 2000” (Reference 4); these entries are also followed by uncertainties in the last digit(s) of the stated values. This uncertainty includes both the estimated measurement uncertainty and the reported range of variation in different terrestrial sources of the element (see Reference 4 for full details and caveats regarding elements whose abundance is variable). The absence of an entry in the Abundance column indicates a radioactive nuclide not present in nature or an element whose isotopic composition varies so widely that a meaningful natural abundance cannot be defined.

References

1. Holden, N. E., “Table of the Isotopes”, in Lide, D. R., Ed., *CRC Handbook of Chemistry and Physics, 86th Ed.*, CRC Press, Boca Raton FL, 2005.
2. Audi, G., Wapstra, A. H., and Thibault, *Nucl. Phys.*, A729, 336, 2003.
3. Audi, G., and Wapstra, A. H., Atomic Mass Data Center, World Wide Web site, <http://www.nndc.bnl.gov/amdc/index.html>
4. de Laeter, J. R., Böhlke, J. K., De Bièvre, P., Hidaka, H., Peiser, H. S., Rosman, K. J. R., and Taylor, P. D. P., *Pure Appl. Chem.* 75, 683, 2003.

Z	Isotope	Mass in u	Abundance in %
1	^{1}H	1.00782503207(10)	99.9885(70)
	^{2}H	2.0141017778(4)	0.0115(70)
	^{3}H	3.0160492777(25)	
2	^{3}He	3.0160293191(26)	0.000134(3)
	^{4}He	4.00260325415(6)	99.999866(3)
3	^{6}Li	6.015122795(16)	7.59(4)
	^{7}Li	7.01600455(8)	92.41(4)
4	^{9}Be	9.0121822(4)	100
5	^{10}B	10.0129370(4)	19.9(7)
	^{11}B	11.0093054(4)	80.1(7)
6	^{11}C	11.0114336(10)	
	^{12}C	12.0000000(0)	98.93(8)
	^{13}C	13.0033548378(10)	1.07(8)
	^{14}C	14.003241989(4)	
7	^{14}N	14.0030740048(6)	99.636(7)
	^{15}N	15.0001088982(7)	0.364(7)
8	^{16}O	15.99491461956(16)	99.757(16)
	^{17}O	16.99913170(12)	0.038(1)
	^{18}O	17.9991610(7)	0.205(14)
9	^{18}F	18.0009380(6)	
	^{19}F	18.99840322(7)	100
10	^{20}Ne	19.9924401754(19)	90.48(3)
	^{21}Ne	20.99384668(4)	0.27(1)
	^{22}Ne	21.991385114(19)	9.25(3)
11	^{22}Na	21.9944364(4)	
	^{23}Na	22.9897692809(29)	100
	^{24}Na	23.99096278(8)	
12	^{24}Mg	23.985041700(14)	78.99(4)
	^{25}Mg	24.98583692(3)	10.00(1)
	^{26}Mg	25.982592929(30)	11.01(3)
13	^{27}Al	26.98153863(12)	100
14	^{28}Si	27.9769265325(19)	92.223(19)
	^{29}Si	28.976494700(22)	4.685(8)
	^{30}Si	29.97377017(3)	3.092(11)
15	^{31}P	30.97376163(20)	100
	^{32}P	31.97390727(20)	
16	^{32}S	31.97207100(15)	94.99(26)
	^{33}S	32.97145876(15)	0.75(2)
	^{34}S	33.96786690(12)	4.25(24)
	^{35}S	34.96903216(11)	
	^{36}S	35.96708076(20)	0.01(1)
17	^{35}Cl	34.96885268(4)	75.76(10)
	^{37}Cl	36.96590259(5)	24.24(10)
18	^{36}Ar	35.967545106(29)	0.3365(30)
	^{38}Ar	37.9627324(4)	0.0632(5)
	^{40}Ar	39.9623831225(29)	99.6003(30)
19	^{39}K	38.96370668(20)	93.2581(44)
	^{40}K	39.96399848(21)	0.0117(1)
	^{41}K	40.96182576(21)	6.7302(44)
	^{42}K	41.96240281(24)	
	^{43}K	42.960716(10)	
20	^{40}Ca	39.96259098(22)	96.941(156)
	^{42}Ca	41.95861801(27)	0.647(23)
	^{43}Ca	42.9587666(3)	0.135(10)
	^{44}Ca	43.9554818(4)	2.086(110)
	^{45}Ca	44.9561866(4)	
	^{46}Ca	45.9536926(24)	0.004(3)
	^{47}Ca	46.9545460(24)	
	^{48}Ca	47.952534(4)	0.187(21)
21	^{45}Sc	44.9559119(9)	100
22	^{46}Ti	45.9526316(9)	8.25(3)
	^{47}Ti	46.9517631(9)	7.44(2)
	^{48}Ti	47.9479463(9)	73.72(3)
	^{49}Ti	48.9478700(9)	5.41(2)
	^{50}Ti	49.9447912(9)	5.18(2)
23	^{50}V	49.9471585(11)	0.250(4)
	^{51}V	50.9439595(11)	99.750(4)
24	^{50}Cr	49.9460442(11)	4.345(13)
	^{51}Cr	50.9447674(11)	
	^{52}Cr	51.9405075(8)	83.789(18)
	^{53}Cr	52.9406494(8)	9.501(17)
	^{54}Cr	53.9388804(8)	2.365(7)
25	^{54}Mn	53.9403589(14)	
	^{55}Mn	54.9380451(7)	100
26	^{52}Fe	51.948114(7)	
	^{54}Fe	53.9396105(7)	5.845(35)
	^{55}Fe	54.9382934(7)	
	^{56}Fe	55.9349375(7)	91.754(36)
	^{57}Fe	56.9353940(7)	2.119(10)
	^{58}Fe	57.9332756(8)	0.282(4)
	^{59}Fe	58.9348755(8)	
27	^{57}Co	56.9362914(8)	

Z	Isotope	Mass in u	Abundance in %
	^{58}Co	57.9357528(13)	
	^{59}Co	58.9331950(7)	100
	^{60}Co	59.9338171(7)	
28	^{58}Ni	57.9353429(7)	68.0769(89)
	^{59}Ni	58.9343467(7)	
	^{60}Ni	59.9307864(7)	26.2231(77)
	^{61}Ni	60.9310560(7)	1.1399(6)
	^{62}Ni	61.9283451(6)	3.6345(17)
	^{63}Ni	62.9296694(6)	
	^{64}Ni	63.9279660(7)	0.9256(9)
29	^{63}Cu	62.9295975(6)	69.15(3)
	^{64}Cu	63.9297642(6)	
	^{65}Cu	64.9277895(7)	30.85(3)
30	^{64}Zn	63.9291422(7)	48.268(321)
	^{65}Zn	64.9292410(7)	
	^{66}Zn	65.9260334(10)	27.975(77)
	^{67}Zn	66.9271273(10)	4.102(21)
	^{68}Zn	67.9248442(10)	19.024(123)
	^{70}Zn	69.9253193(21)	0.631(9)
31	^{67}Ga	66.9282017(14)	
	^{68}Ga	67.9279801(16)	
	^{69}Ga	68.9255736(13)	60.108(9)
	^{71}Ga	70.9247013(11)	39.892(9)
32	^{68}Ge	67.928094(7)	
	^{70}Ge	69.9242474(11)	20.38(18)
	^{72}Ge	71.9220758(18)	27.31(26)
	^{73}Ge	72.9234589(18)	7.76(8)
	^{74}Ge	73.9211778(18)	36.72(15)
	^{76}Ge	75.9214026(18)	7.83(7)
33	^{75}As	74.9215965(20)	100
34	^{74}Se	73.9224764(18)	0.89(4)
	^{75}Se	74.9225234(18)	
	^{76}Se	75.9192136(18)	9.37(29)
	^{77}Se	76.9199140(18)	7.63(16)
	^{78}Se	77.9173091(18)	23.77(28)
	^{79}Se	78.9184991(18)	
	^{80}Se	79.9165213(21)	49.61(41)
	^{82}Se	81.9166994(22)	8.73(22)
35	^{79}Br	78.9183371(22)	50.69(7)
	^{81}Br	80.9162906(21)	49.31(7)
36	^{78}Kr	77.9203648(12)	0.355(3)
	^{80}Kr	79.9163790(16)	2.286(10)
	^{82}Kr	81.9134836(19)	11.593(31)
	^{83}Kr	82.914136(3)	11.500(19)
	^{84}Kr	83.911507(3)	56.987(15)
	^{86}Kr	85.91061073(11)	17.279(41)
37	^{85}Rb	84.911789738(12)	72.17(2)
	^{86}Rb	85.91116742(21)	
	^{87}Rb	86.909180527(13)	27.83(2)
38	^{84}Sr	83.913425(3)	0.56(1)
	^{85}Sr	84.912933(3)	
	^{86}Sr	85.9092602(12)	9.86(1)
	^{87}Sr	86.9088771(12)	7.00(1)
	^{88}Sr	87.9056121(12)	82.58(1)
	^{89}Sr	88.9074507(12)	
	^{90}Sr	89.907738(3)	
39	^{89}Y	88.9058483(27)	100
40	^{90}Zr	89.9047044(25)	51.45(40)
	^{91}Zr	90.9056458(25)	11.22(5)
	^{92}Zr	91.9050408(25)	17.15(8)
	^{94}Zr	93.9063152(26)	17.38(28)
	^{96}Zr	95.9082734(30)	2.80(9)
41	^{93}Nb	92.9063781(26)	100
42	^{92}Mo	91.906811(4)	14.77(31)
	^{94}Mo	93.9050883(21)	9.23(10)
	^{95}Mo	94.9058421(21)	15.90(9)
	^{96}Mo	95.9046795(21)	16.68(1)
	^{97}Mo	96.9060215(21)	9.56(5)
	^{98}Mo	97.9054082(21)	24.19(26)
	^{99}Mo	98.9077119(21)	
	^{100}Mo	99.907477(6)	9.67(20)
43	^{97}Tc	96.906365(5)	
	^{98}Tc	97.907216(4)	
	^{99}Tc	98.9062547(21)	
44	^{96}Ru	95.907598(8)	5.54(14)
	^{98}Ru	97.905287(7)	1.87(3)
	^{99}Ru	98.9059393(22)	12.76(14)
	^{100}Ru	99.9042195(22)	12.60(7)
	^{101}Ru	100.9055821(22)	17.06(2)
	^{102}Ru	101.9043493(22)	31.55(14)
	^{104}Ru	103.905433(3)	18.62(27)
	^{106}Ru	105.907329(8)	
45	^{103}Rh	102.905504(3)	100
46	^{102}Pd	101.905609(3)	1.02(1)
	^{104}Pd	103.904036(4)	11.14(8)
	^{105}Pd	104.905085(4)	22.33(8)
	^{106}Pd	105.903486(4)	27.33(3)
	^{108}Pd	107.903892(4)	26.46(9)
	^{110}Pd	109.905153(12)	11.72(9)
47	^{107}Ag	106.905097(5)	51.839(8)
	^{109}Ag	108.904752(3)	48.161(8)
48	^{106}Cd	105.906459(6)	1.25(6)
	^{108}Cd	107.904184(6)	0.89(3)
	^{110}Cd	109.9030021(29)	12.49(18)
	^{111}Cd	110.9041781(29)	12.80(12)
	^{112}Cd	111.9027578(29)	24.13(21)
	^{113}Cd	112.9044017(29)	12.22(12)
	^{114}Cd	113.9033585(29)	28.73(42)
	^{116}Cd	115.904756(3)	7.49(18)
49	^{111}In	110.905103(5)	
	^{113}In	112.904058(3)	4.29(5)
	^{115}In	114.903878(5)	95.71(5)
50	^{112}Sn	111.904818(5)	0.97(1)
	^{113}Sn	112.905171(4)	
	^{114}Sn	113.902779(3)	0.66(1)
	^{115}Sn	114.903342(3)	0.34(1)
	^{116}Sn	115.901741(3)	14.54(9)
	^{117}Sn	116.902952(3)	7.68(7)
	^{118}Sn	117.901603(3)	24.22(9)
	^{119}Sn	118.903308(3)	8.59(4)
	^{120}Sn	119.9021947(27)	32.58(9)
	^{122}Sn	121.9034390(29)	4.63(3)
	^{124}Sn	123.9052739(15)	5.79(5)
51	^{121}Sb	120.9038157(24)	57.21(5)
	^{123}Sb	122.9042140(22)	42.79(5)
52	^{120}Te	119.904020(10)	0.09(1)
	^{122}Te	121.9030439(16)	2.55(12)
	^{123}Te	122.9042700(16)	0.89(3)
	^{124}Te	123.9028179(16)	4.74(14)
	^{125}Te	124.9044307(16)	7.07(15)
	^{126}Te	125.9033117(16)	18.84(25)
	^{128}Te	127.9044631(19)	31.74(8)

Z	Isotope	Mass in u	Abundance in %
	^{130}Te	129.9062244(21)	34.08(62)
53	^{123}I	122.905589(4)	
	^{125}I	124.9046302(16)	
	^{127}I	126.904473(4)	100
	^{129}I	128.904988(3)	
	^{131}I	130.9061246(12)	
54	^{124}Xe	123.9058930(20)	0.0952(3)
	^{126}Xe	125.904274(7)	0.0890(2)
	^{128}Xe	127.9035313(15)	1.9102(8)
	^{129}Xe	128.9047794(8)	26.4006(82)
	^{130}Xe	129.9035080(8)	4.0710(13)
	^{131}Xe	130.9050824(10)	21.2324(30)
	^{132}Xe	131.9041535(10)	26.9086(33)
	^{134}Xe	133.9053945(9)	10.4357(21)
	^{136}Xe	135.907219(8)	8.8573(44)
55	^{129}Cs	128.906064(5)	
	^{133}Cs	132.905451933(24)	100
	^{134}Cs	133.906718475(28)	
	^{136}Cs	135.9073116(20)	
	^{137}Cs	136.9070895(5)	
56	^{130}Ba	129.9063208(30)	0.106(1)
	^{132}Ba	131.9050613(11)	0.101(1)
	^{133}Ba	132.9060075(11)	
	^{134}Ba	133.9045084(4)	2.417(18)
	^{135}Ba	134.9056886(4)	6.592(12)
	^{136}Ba	135.9045759(4)	7.854(24)
	^{137}Ba	136.9058274(5)	11.232(24)
	^{138}Ba	137.9052472(5)	71.698(42)
	^{140}Ba	139.910605(9)	
57	^{138}La	137.907112(4)	0.090(1)
	^{139}La	138.9063533(26)	99.910(1)
58	^{136}Ce	135.907172(14)	0.185(2)
	^{138}Ce	137.905991(11)	0.251(2)
	^{140}Ce	139.9054387(26)	88.450(51)
	^{141}Ce	140.9082763(26)	
	^{142}Ce	141.909244(3)	11.114(51)
	^{144}Ce	143.913647(4)	
59	^{141}Pr	140.9076528(26)	100
60	^{142}Nd	141.9077233(25)	27.2(5)
	^{143}Nd	142.9098143(25)	12.2(2)
	^{144}Nd	143.9100873(25)	23.8(3)
	^{145}Nd	144.9125736(25)	8.3(1)
	^{146}Nd	145.9131169(25)	17.2(3)
	^{148}Nd	147.916893(3)	5.7(1)
	^{150}Nd	149.920891(3)	5.6(2)
61	^{145}Pm	144.912749(3)	
	^{147}Pm	146.9151385(26)	
62	^{144}Sm	143.911999(3)	3.07(7)
	^{147}Sm	146.9148979(26)	14.99(18)
	^{148}Sm	147.9148227(26)	11.24(10)
	^{149}Sm	148.9171847(26)	13.82(7)
	^{150}Sm	149.9172755(26)	7.38(1)
	^{152}Sm	151.9197324(27)	26.75(16)
	^{154}Sm	153.9222093(27)	22.75(29)
63	^{151}Eu	150.9198502(26)	47.81(6)
	^{153}Eu	152.9212303(26)	52.19(6)
64	^{152}Gd	151.9197910(27)	0.20(1)
	^{154}Gd	153.9208656(27)	2.18(3)
	^{155}Gd	154.9226220(27)	14.80(12)
	^{156}Gd	155.9221227(27)	20.47(9)
	^{157}Gd	156.9239601(27)	15.65(2)
	^{158}Gd	157.9241039(27)	24.84(7)
	^{160}Gd	159.9270541(27)	21.86(19)
65	^{159}Tb	158.9253468(27)	100
66	^{156}Dy	155.924283(7)	0.056(3)
	^{158}Dy	157.924409(4)	0.095(3)
	^{160}Dy	159.9251975(27)	2.329(18)
	^{161}Dy	160.9269334(27)	18.889(42)
	^{162}Dy	161.9267984(27)	25.475(36)
	^{163}Dy	162.9287312(27)	24.896(42)
	^{164}Dy	163.9291748(27)	28.260(54)
67	^{165}Ho	164.9303221(27)	100
68	^{162}Er	161.928778(4)	0.139(5)
	^{164}Er	163.929200(3)	1.601(3)
	^{166}Er	165.9302931(27)	33.503(36)
	^{167}Er	166.9320482(27)	22.869(9)
	^{168}Er	167.9323702(27)	26.978(18)
	^{170}Er	169.9354643(30)	14.910(36)
69	^{169}Tm	168.9342133(27)	100
70	^{168}Yb	167.933897(5)	0.13(1)
	^{169}Yb	168.935190(5)	
	^{170}Yb	169.9347618(26)	3.04(15)
	^{171}Yb	170.9363258(26)	14.28(57)
	^{172}Yb	171.9363815(26)	21.83(67)
	^{173}Yb	172.9382108(26)	16.13(27)
	^{174}Yb	173.9388621(26)	31.83(92)
	^{176}Yb	175.9425717(28)	12.76(41)
71	^{175}Lu	174.9407718(23)	97.41(2)
	^{176}Lu	175.9426863(23)	2.59(2)
72	^{174}Hf	173.940046(3)	0.16(1)
	^{176}Hf	175.9414086(24)	5.26(7)
	^{177}Hf	176.9432207(23)	18.60(9)
	^{178}Hf	177.9436988(23)	27.28(7)
	^{179}Hf	178.9458161(23)	13.62(2)
	^{180}Hf	179.9465500(23)	35.08(16)
73	^{180}Ta	179.9474648(24)	0.012(2)
	^{181}Ta	180.9479958(19)	99.988(2)
74	^{180}W	179.946704(4)	0.12(1)
	^{182}W	181.9482042(9)	26.50(16)
	^{183}W	182.9502230(9)	14.31(4)
	^{184}W	183.9509312(9)	30.64(2)
	^{186}W	185.9543641(19)	28.43(19)
75	^{185}Re	184.9529550(13)	37.40(2)
	^{187}Re	186.9557531(15)	62.60(2)
76	^{184}Os	183.9524891(14)	0.02(1)
	^{186}Os	185.9538382(15)	1.59(3)
	^{187}Os	186.9557505(15)	1.96(2)
	^{188}Os	187.9558382(15)	13.24(8)
	^{189}Os	188.9581475(16)	16.15(5)
	^{190}Os	189.9584470(16)	26.26(2)
	^{192}Os	191.9614807(27)	40.78(19)
77	^{191}Ir	190.9605940(18)	37.3(2)
	^{193}Ir	192.9629264(18)	62.7(2)
78	^{190}Pt	189.959932(6)	0.014(1)
	^{192}Pt	191.9610380(27)	0.782(7)
	^{194}Pt	193.9626803(9)	32.967(99)
	^{195}Pt	194.9647911(9)	33.832(10)
	^{196}Pt	195.9649515(9)	25.242(41)
	^{198}Pt	197.967893(3)	7.163(55)
79	^{197}Au	196.9665687(6)	100
	^{198}Au	197.9682423(6)	
80	^{196}Hg	195.965833(3)	0.15(1)

Z	Isotope	Mass in u	Abundance in %
	^{197}Hg	196.967213(3)	
	^{198}Hg	197.9667690(4)	9.97(20)
	^{199}Hg	198.9682799(4)	16.87(22)
	^{200}Hg	199.9683260(4)	23.10(19)
	^{201}Hg	200.9703023(6)	13.18(9)
	^{202}Hg	201.9706430(6)	29.86(26)
	^{203}Hg	202.9728725(18)	
	^{204}Hg	203.9734939(4)	6.87(15)
81	^{201}Tl	200.970819(16)	
	^{203}Tl	202.9723442(14)	29.52(1)
	^{205}Tl	204.9744275(14)	70.48(1)
82	^{204}Pb	203.9730436(13)	1.4(1)
	^{206}Pb	205.9744653(13)	24.1(1)
	^{207}Pb	206.9758969(13)	22.1(1)
	^{208}Pb	207.9766521(13)	52.4(1)
	^{210}Pb	209.9841885(16)	
83	^{207}Bi	206.9784707(26)	
	^{209}Bi	208.9803987(16)	100
84	^{209}Po	208.9824304(20)	
	^{210}Po	209.9828737(13)	
85	^{210}At	209.987148(8)	
	^{211}At	210.9874963(30)	
86	^{211}Rn	210.990601(7)	
	^{220}Rn	220.0113940(24)	
	^{222}Rn	222.0175777(25)	
87	^{223}Fr	223.0197359(26)	
88	^{223}Ra	223.0185022(27)	
	^{224}Ra	224.0202118(24)	
	^{226}Ra	226.0254098(25)	
	^{228}Ra	228.0310703(26)	
89	^{227}Ac	227.0277521(26)	
90	^{228}Th	228.0287411(24)	
	^{230}Th	230.0331338(19)	
	^{232}Th	232.0380553(21)	100
91	^{231}Pa	231.0358840(24)	100
92	^{233}U	233.0396352(29)	
	^{234}U	234.0409521(20)	0.0054(5)
	^{235}U	235.0439299(20)	0.7204(6)
	^{236}U	236.0455680(20)	
	^{238}U	238.0507882(20)	99.2742(10)
93	^{237}Np	237.0481734(20)	
	^{239}Np	239.0529390(22)	
94	^{238}Pu	238.0495599(20)	
	^{239}Pu	239.0521634(20)	
	^{240}Pu	240.0538135(20)	
	^{241}Pu	241.0568515(20)	
	^{242}Pu	242.0587426(20)	
	^{244}Pu	244.064204(5)	
95	^{241}Am	241.0568291(20)	
	^{243}Am	243.0613811(25)	
96	^{243}Cm	243.0613891(22)	
	^{244}Cm	244.0627526(20)	
	^{245}Cm	245.0654912(22)	
	^{246}Cm	246.0672237(22)	
	^{247}Cm	247.070354(5)	
	^{248}Cm	248.072349(5)	
97	^{247}Bk	247.070307(6)	
	^{249}Bk	249.0749867(28)	
98	^{249}Cf	249.0748535(24)	
	^{250}Cf	250.0764061(22)	
	^{251}Cf	251.079587(5)	
	^{252}Cf	252.081626(5)	
99	^{252}Es	252.082980(50)	
100	^{257}Fm	257.095105(7)	
101	^{256}Md	256.094060(60)	
	^{258}Md	258.098431(5)	
102	^{259}No	259.10103(11)*	
103	^{262}Lr	262.10963(22)*	
104	^{261}Rf	261.108770(30)*	
105	^{262}Db	262.11408(20)*	
106	^{263}Sg	263.11832(13)*	
107	^{264}Bh	264.12460(30)*	
108	^{265}Hs	265.13009(15)*	
109	^{268}Mt	268.13873(34)*	
110	^{281}Ds	281.16206(78)*	
111	^{272}Rg	273.15362(36)*	
